# Effect of a self-care educational intervention to improve self-care adherence among patients with chronic heart failure: a clustered randomized controlled trial in Northwest Ethiopia

**DOI:** 10.1186/s12872-021-02170-8

**Published:** 2021-08-03

**Authors:** Getenet Dessie, Sahai Burrowes, Henok Mulugeta, Dessalegn Haile, Ayenew Negess, Dubie Jara, Girma Alem, Bekele Tesfaye, Haymanot Zeleke, Tenaw Gualu, Temsgen Getaneh, Getiye Dejenu Kibret, Desalegne Amare, Endalkachew Worku Mengesha, Fasil Wagnew, Rasheda Khanam

**Affiliations:** 1 Department of Nursing, School of Health Science, College of Medicine and Health Science, Bahir Dar University, Bahir Dar, Ethiopia; 2 Public Health Program, College of Education and Health Sciences, Touro University California, Vallejo, USA; 3 Departments of Nursing, College of Health Science, Debre Markos University, Debre Markos, Ethiopia; 4 Departments of Human Nutrition and Food Science, College of Health Science, Debre Markos University, Debre Markos, Ethiopia; 5 Department of Public Health, College of Health Science, Debre Markos University, Debre Markos, Ethiopia; 6 Department of Midwifery, College of Health Science, Debre Markos University, Debre Markos, Ethiopia; 7 Australian Centre for Public and Population Health Research, School of Public Health, Faculty of Health, University of Technology Sydney, Ultimo, NSW Australia; 8 Department of Reproductive Health and Population Studies, School of Public Health, College of Medicine and Health Science, Bahir Dar University, Bahir Dar, Ethiopia; 9 School of Commerce, Centre for Health Research, University of Southern Queensland, Toowoomba City, Australia

**Keywords:** Heart failure, Self-care adherence, Self-care education, Ethiopia

## Abstract

**Background:**

As the burden of cardiovascular disease increases in sub-Saharan Africa, there is a growing need for low-cost interventions to mitigate its impact. Providing self-care health education to patients with chronic heart failure (CHF) is recommended as an intervention to prevent complications, improve quality of life, and reduce financial burdens on fragile health systems. However, little is known about health education’s effectiveness at improving CHF self-management adherence in sub-Saharan Africa. Therefore the present study aimed to assess the effectiveness of an educational intervention to improve self-care adherence among patients with CHF at Debre Markos and Felege Hiwot Referral Hospitals in Northwest Ethiopia.

**Methods:**

To address this gap, we adapted a health education intervention based on social cognitive theory comprising of intensive four-day training and, one-day follow-up sessions offered every four months. Patients also received illustrated educational leaflets. We then conducted a clustered randomized control trial of the intervention with 186 randomly-selected patients at Debre Markos and Felege Hiwot referral hospitals. We collected self-reported data on self-care behavior before each educational session. We analyzed these data using a generalized estimating equations model to identify health education's effect on a validated 8-item self-care adherence scale.

**Results:**

Self-care adherence scores were balanced at baseline. After the intervention, patients in the intervention group (n = 88) had higher adherence scores than those in the control group (n = 98). This difference was statistically significant (β = 4.15, *p* < 0.05) and increased with each round of education. Other factors significantly associated with adherence scores were being single (β = − 0.25, *p* < 0.05), taking aspirin (β = 0.76, *p* < 0.05), and having a history of hospitalization (β = 0.91, *p* < 0.05).

**Conclusions:**

We find that self-care education significantly improved self-care adherence scores among CHF patients. This suggests that policymakers should consider incorporating self-care education into CHF management.

*Trial registration number*: PACTR201908812642231

**Supplementary Information:**

The online version contains supplementary material available at 10.1186/s12872-021-02170-8.

## Introduction

### Background

The burden of cardiovascular diseases (CVD) is increasing rapidly in sub-Saharan Africa (SSA) [1]. Between 1990 and 2017, the burden of CVD, as measured by all-age disability-adjusted life years (DALYs), increased by 47% in the region [2] and, in 2013, CVD accounted for approximately one million deaths (approximately 11% of the region’s deaths that year) [3]. The rising burden of CVD is thought to be driven by increased exposure to risk factors such as high fat and high sodium diets, air pollution, tobacco smoke, and aging populations [3]. Increasing CVD due to these new exposures occurs on top of historically high CVD levels caused by infection-related rheumatic disease [4].

As the burden of CVD continues to increase in SSA, so too will the rate of complications such as chronic heart failure (CHF). Heart failure is a complex clinical syndrome characterized by a structural or functional cardiac disorder that impairs the ventricle’s ability to fill or pump blood [5]. It develops when the heart’s pumping ability is insufficient to maintain adequate tissue perfusion [6, 7]. Several risk factors such as ischemic heart disease, hypertension, smoking, obesity, diabetes increase heart failure incidence and severity [8].

Increasing heart failure rates in SSA are concerning because heart failure often leads to reoccurring hospitalization. In SSA, despite important therapeutic advances and national quality improvement efforts, its long-term mortality rate remains high and has improved little over time [9, 10]. For example, literature review of heart failure in African patients with diabetes found a case-fatality rate that ranged from 9 to 12.5% [11]. High rates of hospitalizations, readmissions, and outpatient visits linked to heart failure are estimated to cost the global health system more than $108 billion annually [12–14].

One reason for the poor health outcomes experienced by CHF patients is the need for ongoing post-acute care that involves patients adopting and maintaining a complex array of behavioral changes. These include stopping smoking, limiting alcohol intake, eating a low-sodium diet, exercising, monitoring symptoms, and taking prescribed medication on schedule [15]. Adopting these “self-care” behaviors can be overwhelming to patients, and adherence is often poor [16]. In Ethiopia, several studies have found poor self-care management practices among CHF patients [17–19] and the country has high CHF-related hospital admissions and deaths [20, 21], with poor medication adherence being the major risk factor [21]. Unlike other common chronic diseases such as diabetes and HIV, there are no policies for systematically providing self-care instructions to CHF patients’ in Ethiopia [22]. This lack of patient support is worrying because ongoing, CHF-related illness, hospitalizations, and readmissions can have devastating impacts on patients’ quality of life and place significant social and economic strains on patients, their families, and their communities [23].

A common intervention to promote self-care is patient self-care education programs [24, 25]. Such programs aim not only to encourage patients to monitor symptoms, take medication, and adhere to behavioral guidelines, but also to empower patients to make decisions about when further care is needed and to engage them as active partners with healthcare providers in managing their care [26, 27]. Self-care educational interventions have been shown to increase CHF patients’ adherence to self-care recommendations, reduce hospital readmissions, and improve CHF patient’s functional status and quality of life [28–30]. Heart failure patients who attend educational sessions have been shown to have lower morbidity and mortality risk than those who did not [31]. Sex, moderate to severe comorbidity, depression, and self-care confidence are significantly associated with patients’ adherence to adopting these “self-care” recommendations [13, 27, 32].

The potential for self-care interventions to reduce hospital readmission is especially significant in strained health systems such as Ethiopia’s that are struggling to address both highly prevalent communicable diseases and growing rates of non-communicable diseases. The cost-effectiveness of having patients strictly adhere to hospital-or home-based self-care management programs is well-established in the scientific literature [33]. Overall, the epidemiological transition in low-income countries away from infectious diseases, towards chronic diseases that require lifelong therapy, requires policymakers to emphasize self-care management systems for chronic diseases [34, 35].

Although CHF self-management health education is a well-established intervention in high-income countries, relatively few rigorous studies of such educational programs have been conducted in sub-Saharan Africa. Therefore, this cluster randomized control trial (RCT) aims to investigate the effectiveness of an educational intervention aimed at improving self-care adherence among patients with chronic heart failure in a low-income, peri-urban setting. The finding of this study will lay the groundwork for expanding such programs in the study area and nationally.

## Methods

### Study area and period

The study was conducted from November 1, 2018, to November 1, 2019, in two public referral hospitals located in the Amhara region of Ethiopia: Debre Markos Referral Hospital and Felege Hiwot Referral Hospital. The hospitals are based in two of the region’s urban centers, but the patient populations are primarily rural agricultural workers and homemakers from the surrounding countryside. Approximately 20,000 patients are admitted to these facilities each year, where they are served by 576 healthcare providers (including 316 nurses, 140 midwives, and 112 physicians). Both hospitals have chronic follow-up clinics attended by more than 3,000 CHF patients annually.

### Study design and eligibility

We used a two-arm, parallel, clustered-randomized control trial to assess a self-care educational intervention's effectiveness on patient self-care adherence scores. All adult patients with a diagnosis of CHF (diagnosed either by imaging or by New York Heart Association (NYHA) functional Class I–IV) [36, 37] who were also conscious and literate were eligible for recruitment. Patients with end-stage heart failure and those transferred from other hospitals were excluded from the study.

### Sample size determination

We calculated our sample size using the following assumptions, which were based on the findings of a similar Taiwanese study that compared the difference between two mean scores [28]: a 95% confidence interval (2-sided); 80% power; a sample size ratio of 1 between control and treatment group, and an expected mean and standard deviation of 65.5 ± 12.2 for the control group and 72.5 ± 11.31 for the treatment group. Based on these assumptions, the sample size required per group was 45. However, because our study randomizes an intervention over facility clusters rather than individual participants, the sample size was adjusted using an intra-cluster correlation coefficient to calculate a design effect. We selected an intraculster correlation coefficient of (ρ) = 0.035 from a study of such coefficients in cardiovascular disease prevention and management in primary care settings [38], and assumed a cluster size of 2. Using the following formula for calculating a design effect, (DE) of 1 + ρ(m − 1), where “m” is the number of subjects in a cluster, we found a design effect of 2.54; a required sample size of 114 patients for each study arm (45*2.54); and final total sample size of 228 patients.

### Randomization of the intervention

We used a cluster randomization strategy to minimize the risk of information contamination. Using a coin flip, Debre Markos Referral Hospital was randomly selected to be the intervention facility, and Felge Hiwot Referral Hospital was selected to be the control facility. Within each facility, patients were selected for inclusion using a simple random sampling technique. We created numbered, ordered lists of all CHF patient identification codes (IDs) at each facility, with the number 1 for the first patient ID, 2 for the second, and so forth. We then created a computer-generated list of random numbers. Patients whose numbered place on the patient ID list corresponded to a number on the random number list were selected to participate in the study.

### Recruitment

Patients were recruited either in person, immediately after receiving treatment at the follow-up clinic, or by phone, using the phone numbers provided on medical records. We obtained written informed consent from all patients before enrollment.

### Study variables and measurement of the outcome variable

The study outcome variable was a heart failure self-care adherence score measured by the eight-item validated Medical Outcomes Study-Specific Adherence Scale (MOS-SAS) [39]. The self-care behaviors assessed by the scale are (1) regular exercise, (2) taking medication as prescribed, (3) consuming one or fewer alcoholic beverage per day, (4) cutting down on smoking or not smoking, (5) following a low salt diet, (6) following a low-fat diet, (7) weighing oneself daily, and (8) monitoring and paying attention to symptoms. We validated the scale for the Ethiopian context using principal components analysis with oblique (Promax) rotation to identify underlying factors in the scale. We retained all eight items as they had Eigenvalues ranging from 1.80 to 3.16 and factor loadings greater than 0.40. The eight-item scale was found to be internally consistent, having a Cronbach’s alpha of 0.94.

For each item on the scale, participants were asked to rate how often they had carried out a particular activity in the past month. They could choose responses on a Likert scale ranging from 0 to 5 (0 “none of the time”, 0 “a little of the time”, 1 “some of the time”, 2 “a good bit of the time”, 4 “most of the time”, or 5 “all of the time”). Each respondent’s total score was calculated by summing responses to all 8 questions; scores could range from 0 to 40. We controlled for socio-demographic, socio-economic characteristics in our models and included both self-reported and health record data on respondents’ medical characteristics as potential explanatory variables. Medical characteristics included a depression score calculated from the nine-item Patient Health Questionnaire-9 (PHQ-9) scale [40]. The scale was found to be valid in the study context, with all items having factor loading greater than 0.40; and internally consistent with a Cronbach alpha of 0.81 (Additional file 1).

### Intervention (self-care education)

The study employed a cluster-level, hospital-based educational intervention. At the intervention facility, a randomly selected CHF patient group received education about self-care. At the control facility, a randomly selected group of patients received usual care and were followed by the project.

The education session curriculum was developed based on CHF management guidelines produced by the American Heart Failure Society and the Ethiopian Ministry of Health [41–43]. The educational sessions covered recognizing HF, adhering to prescription medication, following dietary guidelines, compiling with a low-salt diet, limiting alcohol intake, maintaining a low-fat diet, exercising, and smoking cessation. Drawing on Social Cognitive Theory [44], the project team developed a series of visual presentations that included videos, photographs, and illustrated handouts as well as interactive exercises in which participants could share experiences and learn by observing others. The face validity of educational materials was determined in a review by a panel of ten experts consisting of physicians, nutritionists, nurses, health educators, and physiotherapists.

The educational sessions were led by Debre Markos Referral Hospital nursing staff and took place at the facility. Each session lasted approximately one hour and was attended by groups of up to 10 people at a time. At the beginning of the study, patients in the treatment group received four educational sessions over four consecutive days. They then received a one-hour follow-up educational session every four months until the end of the project, for six educational sessions in total over the one-year project period. Project nurses collected self-adherence data at the start of the study for a baseline and before each follow-up education session. This information A printed educational leaflet was provided for each treatment participant to aid in-home care after completing the initial educational sessions.

### Usual care

Participants in the control group received the usual care customarily administered to HF patients receiving treatment from the hospital. This involved providing patients with basic discharge instructions.

### Data collection and quality control

A structured questionnaire was prepared in English, and then translated to Amharic and then back to English by a professional translator to check for consistency of the questions and comparability of the findings. Pre-tests of the data collection tools were conducted with 12 patients (5% of the sample size) from a population with similar characteristics at Gondar Referral Hospital. Before the data collection, we made necessary corrections based on analyzing this pre-test data.

The project collected comprehensive baseline data on patent demographics, socio-economic status, medical history, laboratory results, and self-care adherence. Every four months, the patients were assessed for self-care adherence, medication changes, new procedures, new diagnoses, and hospitalizations either by phone or in person at educational sessions. We collected mortality data through medical record review and information from family members. Hospitalization data were obtained from medical records review, outpatient notes from any specialist encounter for any admission to an outside hospital, and direct patient inquiry during follow-up.

### Data processing and analysis procedure

The collected data were checked for completeness and inconsistencies, then entered into EpiData version 3.1 and exported to Stata version 14 for analysis. We described important study variables using frequencies, percents, and means. To identify the impact of health education on self-care adherence, we fitted a generalized estimating equations (GEE) model that takes into account clustering and within-subject correlation due to the fact that our data contained repeated measures within facilities. We choose the working correlation matrix for the GEE model using the quasi-likelihood under the independence model information criterion (QIC) for model selection [45] to evaluate which matrix the data best. We selected an autoregressive correlation structure as it had the lowest QIC and small standard errors on parameter estimates. We considered beta coefficients with *p* values ≤ 0.05 and corresponding 95% CI statistically significant.

## Results

### Socio-demographic characteristics

**Fig. 1:**
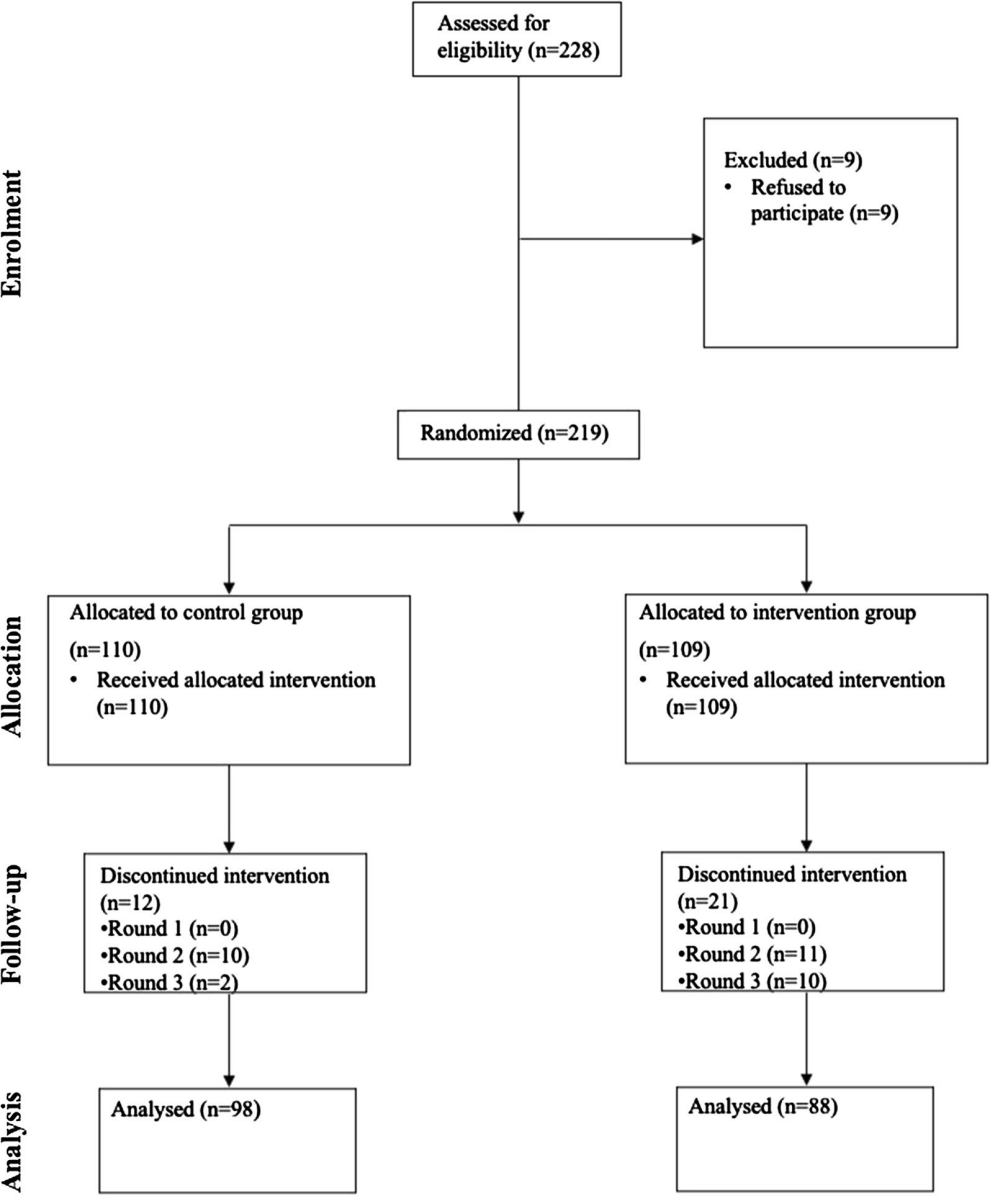
Flow of patients through the study (CONSORT Diagram) to assess the effect of health education on CHF patients’ mean self-care adherence score in Debre Markos and Feleg Hiwot referral hospitals, 2018/2019

The final estimation study sample consisted of 186 participants (81.6% response rate). Of the 228 respondents recruited, 219 agreed to participate, and 33 were subsequently lost to follow up (LTFU). In the control arm, 10 were LTFU during the second round of education sessions, and two were LTFU in the third round. In the treatment arm, 11 and 10 participants were LTFU in the second and third rounds of education sessions, respectively (see Fig. 1).

Table 1 reports the baseline characteristics of the control and treatment groups. The groups were unbalanced on several socio-demographic characteristics at baseline. These variables were, therefore, included in our regression analysis as possible confounders. For example, there were significant differences in age between the control and treatment groups. The median age was 50 years (IQR: 30–60) in the control group and 37.5 years (IQR: 25–50.75) in the treatment group. Similarly, more than two-thirds (72.4%) of the control group lived in urban areas compared to only one-third (37.5%) in the treatment group. Participants in the control group were also significantly more likely to be female and less likely to be single. The majority of participants in both groups only had less than a secondary school level of education, and most worked as farmers or homemakers.

**Table 1:** Socio-demographic characteristics of CHF patients at Debre Markos Referral Hospital and Feleg Hiwot Referral Hospital, Ethiopia, 2018/19 (n = 186)

Variables	Control group (98)	Treatment group (n = 88)	*p* value
	Frequency (%)	Frequency (%)	
*Sex*			
Male	31(31.6%)	46(52.3%)	*p* < 0.0001
Female	67(68.4%)	42 (47.7%)	
*Residence*			
Urban	71 (72.4%)	33 (37.5%)	*p* < 0.0001
Rural	27 (27.6%)	55 (62.5%)	
*Marital status*			
Single/separated	21 (21.4%)	29 (32.9%)	*p* < 0.0001
Married	58 (59.2%)	51 (58%)	
Divorced	15(15.3%)	-	
Widowed	4 (4.1%)	8 (9.1%)	
*Educational status*			
Cannot read and write	55(56.1%)	38 (43.2%)	*p* = 0.37
Primary school	28(28.6%)	33 (37.5%)	
Secondary school	8(8.2%)	6 (6.8%)	
Diploma	5(5.1%)	7 (8%)	
Degree	2(2%)	4 (4.5%)	
*Religion*			
Orthodox	91 (92.9%)	82 (93.2%)	*p* = 0.93
Others*	7 (6.1%)	6 (6.8%)	
*Occupation*			
Farmer	36 (36.7%)	37 (42%)	*p* = 0.23
Homemaker	38 (38.8%)	22 (25%)	
Government employee	9 (9.2%)	13 (14.8%)	
Student	7 (7.1%)	5 (5.7%)	
*Source of family support*			
None	8 (8.1%)	2 (2.3%)	*p* = 0.08
Family	90 (91.9%)	86 (97.7%)	

*Muslim or Protestant

### Medical characteristics of participants

The mean (± SD) duration of time since CHF diagnosis for the control and treatment groups was 4.1 (± 12) and 4.6 (± 7.6) years, respectively. Control group participants had a significantly higher baseline weight (67.8 kg ± 6.03) than the treatment group (60.2 kg ± 10.4). Significantly fewer participants in the control group had comorbidities compared to those in the treatment group (17.3% compared to 36.4%) but control group participants had more severe CHF ratings. Most participants had a previous hospital admission: 68.4% from the control group and 80.7% from the treatment group (see Table 2).

**Table 2:** Clinical characteristics of participants receiving care at Debre Markos and Felege Hiwot referral hospitals, Ethiopia, 2018–2019 (n = 186)

Variables	Control group (n = 98)	Treatment group (n = 88)	*p* values
	Frequency (%)	Frequency (%)	
*Comorbidity*			
Yes	17 (17.3%)	32 (36.4%)	0.000
No	81 (82.7%)	56 (63.6%)	
*Current medication*			
Furosemide	87 (88.8%)	88 (100%))	0.000
Spironolactone	76 (77.6%)	83 (94.3%)	
ACE	42 (42.8%)	55 (62.5%)	
Aspirin	44 (44.9%)	12 (13.6%)	
Lipid lowering drugs	18 (18.3%)	12 (13.6%)	
Beta blockers	51 (52%)	44 (50%)	
*History of hospitalization*			
Yes	67 (68.4%)	71 (80.7%)	0.06
No	31 (31.6%)	17 (19.3%)	
*Chronic heart failure stage*			
One	–	5 (5.7%)	0.000
Two	3 (3.1%)	14 (15.9%)	
Three	19 (19.4%)	21 (23.9%)	
Four	76 (77.6%)	48 (54.5%)	

ACE: Angiotensin converting enzyme inhibitors. In this study ACE inhibitors were captopril and enalapril

Beta blockers were atenolol, metoprolol, and propranolol

### Adherence score among control and treatment group

**Fig. 2:**
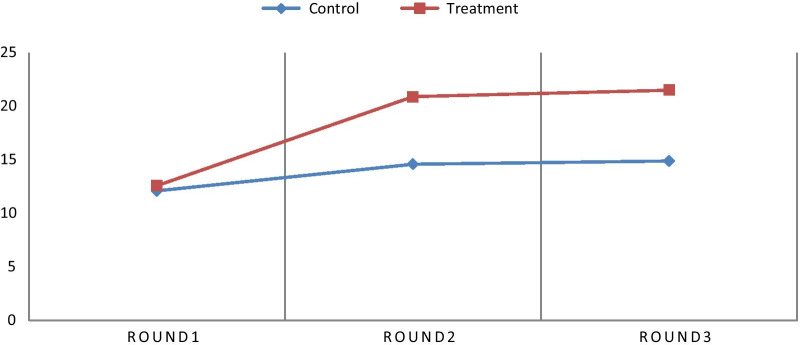
CHF patients’ mean self-care adherence score in Debre Markos and Feleg Hiwot referral hospitals, 2018/2019

At baseline there was no significant difference between the control and treatment groups in self-care adherence scores: the mean (± SD) baseline adherence score of the control group was 12.1 points (± 2.8) and 12.6 points (± 2.5) for the treatment group (*p* = 0.998 in two-tailed t-tests). At the second round of health education, the control group’s mean adherence score increased to 14.6 points ± 2.7 and the treatment group’s score to 20.9 points (± 7.04). At the third round of health education, adherence scores remained roughly constant, with the mean control group score of 14.9 points ± 2.1 and a treatment group score of 21.5 ± 1.9 (see Fig. 2). The difference in the increase in the mean adherence score between treatment and control groups after baseline was statistically significant (*p* = 0.024).

### Factors affecting adherence score of self-care recommendations

We found five variables to be significantly and independently associated with adherence to self-care implementation in our final generalized estimating equation model across most correlation structure specifications (see Table 3). Being in the treatment group had a large, positive effect on the self-care adherence scores when controlling other socio-demographic and medical factors (β = 4.15, *p* < 0.05). In other words, receiving treatment increased the self-care adherence score by 4.15 points. There was a statistically significant interaction between being in treatment and the number of educational sessions attended, implying that impact of the treatment was stronger in those who attended multiple sessions (β = 7.14, *p* < 0.001).

**Table 3:** Estimates of the effect of predictor variables on self-care adherence score among CHF patients, Northwest Ethiopia 2018/2019

Adherence	Beta	Std. error	95% Confidence interval		Significance level
			Lower	Upper	
Treatment	4.151	0.492	3.186	5.115	0.001
Age in years	0.006	0.009	− 0.011	0.023	0.470
Sex (male = 1)	− 0.223	0.224	− 0.662	0.217	0.321
Single/separated	− 0.250	0.126	− 0.497	− 0.002	0.048
Support	0.599	0.498	− 0.378	1.576	0.229
Taking aspirin (yes = 1)	0.757	0.306	0.157	1.358	0.013
Depression score	− 0.706	0.375	− 1.442	0.029	0.052
Weight	0.069	0.043	− 0.015	0.152	0.107
Prior hospitalization (yes = 1)	0.905	0.394	0.134	1.677	0.021
Number of sessions attended	1.411	0.202	1.014	1.807	0.001
Treatment * sessions	7.142	0.236	6.679	7.604	0.001
(Intercept)	9.981	2.208	5.653	14.309	0.001

Being separated /single reduced the self-care adherence score by 0.25 points (*p* < 0.05). Patients with a history of hospitalization (β = 0.905, *p* < 0.05) and those who were taking aspirin (β = 0.757, *p* < 0.05) also had slightly higher self-care adherence scores when controlling for socio-demographic and medical characteristics. Finally, the number of health education sessions attended increased self-care adherence scores on average by 1.41 points for each visit (Table 3).

## Discussion

This study assessed the effect of a self-care education intervention on self-care adherence among CHF patients in Northwest Ethiopia. We found that the intervention was associated with a statistically significant, four-point increase on our 40-point self-care adherence scale. This finding is in keeping with the results of similar experimental studies of CHF self-care programs conducted in Taiwan [28] and Turkey [30]. Self-care health education programs for other chronic diseases, such as hypertension, have also been found to improve disease control [46]. Surveys of CHF self-management in Ethiopia have consistently found that greater knowledge of heart failure and its management is positively associated with self-care adherence; suggesting that improving patients’ understanding of CHF and the importance of self-care can act as a catalyst, encouraging HF patients to fully engage in their care [47–49].

Our findings and those of previous studies have important policy implications because they suggest that by developing well-structured patient education programs to increase self-care adherence, Ethiopia could mitigate CHF’s impact on quality of life, and reduce the country’s high CHF-related mortality and hospital readmission rates even in rural populations with limited formal education [31].

Our findings on the factors associated with self-care adherence are in keeping with previous studies on the topic. For example, our finding that being separated or divorced was associated with a small but significant reduction in the self-care adherence score aligns with studies that have found unmarried patients more likely to be non-adherent than married patients [40] and less likely to have good social support or to be in good physical health [41].

Social support itself was not associated with self-care adherence in our study, perhaps because there was so little variation in reported levels of social support in our sample. However, studies often use marriage as a proxy for social support, and social support, in turn, is consistently associated with reduced cardiovascular disease and better health-seeking and self-care behaviors such as exercising regularly, taking flu shots, and limiting fluid intake [42, 43]. The significance of marital status in our models underscores family-focused educational interventions’ suitability to improve self-care behavior. Education programs that actively involve patients’ family members have been shown to be effective in Ethiopian patients with heart failure [44, 45].

A history of previous hospitalization increased self-care adherence in our study. Previous systematic reviews and meta-analyses have also found that patients who had been institutionalized in the past were more adherent to their medication than those who had not [46]. In Ethiopia, this phenomenon may be explained by the discharge instructions that Ethiopian healthcare facilities must give to patients with chronic illness [47] since receiving discharge instructions is believed to increase adherence to self-care recommendations [48]. The association between previous hospitalization and self-care highlights the potential for systematic, pre-discharge patient education programs to improve patient self-care adherence. Nurses and physicians currently provide such structured education programs for diabetic patients, but discharge education for CHF patients is unstructured and ad hoc [38]. Adopting ongoing, structured training for CHF patients could improve adherence and minimize the economic burden that comes from readmission [49, 50].

We found that patients who were taking aspirin had higher adherence scores than those who were not. According to Ethiopian national treatment guidelines for CHF patients, low-dose aspirin therapy is prescribed for high-risk groups based on their lipid profile and for the elderly to reduce coagulopathy risk. Most importantly, aspirin is administered to those with a previous history of strokes. As mentioned above, patients with a history of coronary heart disease often adhere to prescribed drugs to prevent a second coronary heart disease event [51]. Our study’s high adherence scores among aspirin takers may, therefore, not result from the drug itself, but rather may be due to special adherence instructions given to high-risk groups.

Finally, we find that the number of educational sessions attended had a significant impact on self-care adherence scores. As Fig. 2 shows, adherence scores increase after the initial session. This finding is consistent with past studies [50]. We designed our educational intervention based on social cognitive theory’s emphasis on a person’s experiences, and assumption that a person’s behavior is shaped by continuous education [51]. Social Cognitive Theory assumes that with continuous education and health promotion, a patient can develop awareness and effective practical skills [52]. This might explain the dramatic increase in the adherence scores from visit to visit among intervention groups.

However depression is not significantly associated with adherence score. A previous study has also reported that absence of significant relationship between overall reported self-care adherence and depression [53]. Similarly in Tang et al.study despite there was a significant difference between depressed and no depressed participants in self-reported medication adherence, there was no difference in objectively measured medication adherence score [54]. This shows that unless we designed special health education to decrease depression itself, educating patients about self-care adherence alone could not bring significant declined in depression score as it has been shown in our finding. In our finding depression score was not significantly varied among treatment and control groups through the study period. This finding suggests that the need of integrating psycho social education to decease depression along with self-care education for CHF patients.

### Study strengths and limitations

The study’s strengths include its rigorous design, its theory-informed intervention, and its use of validated, pre-tested instruments. Among its limitations is our self-care adherence measure’s self-reported nature. It is reasonable to assume that at least some of the increase in self-care adherence scores that we found may have resulted from social desirability bias. Secondly, the study was small and under our target sample size. It was conducted at only two hospitals, with randomization occurring at the hospital-level. Although the hospitals had similar patient populations and overlapping catchment areas, unobserved differences in the hospitals’ patient populations would have persisted under our randomization strategy. Facility-level differences may explain the imbalance in socio-demographic and medical characteristics between the study arms at baseline. We took this imbalance into account by adjusting for these factors in our multivariable analysis.

## Conclusion

A self-care education program based on social cognitive theory appears to be effective in increasing self-care adherence scores of patients with CHF in a low-income setting in Ethiopia. Marital status, taking aspirin, a history of hospitalization, and the number of educational sessions attended were significantly associated with patients’ adherence scores. Our findings suggest that the Ethiopian Ministry of Health should explore incorporating structured self-care education programs into the routine care of people with CHF.

## Supplementary Information

**Additional file 1**: Information sheet and data collection tool to assess the effectiveness of an educational intervention to improve self-care adherence among patients with chronic heart failure at Debre Markos and Felege Hiwot Referral Hospitals in Northwest Ethiopia, 2018/2019**.**

## Data Availability

The study datasets are available from the corresponding author and will be shared upon reasonable request.

## Abbreviations

CHF: Chronic heart failure
CVD: Cardiovascular diseases
RCT: Randomized control trial
SD: Standard deviation
SSA: Sub-Saharan Africa
