# Supplementary material for: Effect of a self-care educational intervention to improve self-care adherence among patients with chronic heart failure: a clustered randomized controlled trial in Northwest Ethiopia
Source: BMC Cardiovasc Disord. 2021 Aug 3;21:374. doi: 10.1186/s12872-021-02170-8 (PMC8336108; doi:10.1186/s12872-021-02170-8)
Supplement: Supplementary file 1 — Additional file 1: Information sheet and data collection tool to assess the effectiveness of an educational intervention to improve self-care adherence among patients with chronic heart failure at Debre Markos and Felege Hiwot Referral Hospitals in Northwest Ethiopia, 2018/2019. [file 12872_2021_2170_MOESM1_ESM.docx]

# **ANNEX I: Information Sheet for Study Subjects**

You are kindly invited to participate in this study, which involves chronic heart failure patients.

**A. Purpose:** The purpose of this study is to assess the impact of Self-Care education on self-care adherence among chronic heart failure patients attending at Debre Markos and Felege hiwot referral hospital 2018/2019**.**

**B. Duration:** The duration of this study is from November 1, 2018, to November 1, 2019

**C. Procedures to be carried on:** the procedure of data collection is easy and straight forward; questionnaire concerning your socio-demographic characteristics, medical history, level of depression and level of Adherence to Heart Failure Self-Care Recommendations is listed. Therefore you are kindly requested to answer accordingly.

**D. Risk and discomfort:** There is no harm of any degree associated with participating in this study, other than your precious time.

**E. Expected benefits**: You will not get special and direct benefit from this study. However, your participation in this study will help in identifying the impact of Self-Care education on self-care adherence and this will in turn result in identifying key statistics and patterns related to Adherence to Heart Failure Self-Care Recommendations and it also used as a base line data for policy reviewers.

**G. Confidentiality:** All your personal information collected for the purpose of the present study will be held in strict confidentiality.

**H. Compensation:** No compensation will be provided by participating in this study.

**I. Termination of the study:** Participation in the study is voluntary, and refusal to participate involves no penalty or loss of benefits to which you are otherwise entitled. The study participants have a right to;

- - Keep hold information
  - Decline to cooperate in the study
  - To refuse provision of data

But your genuine answer is more than important for this study as well as for credible finding.

We would also like to inform you that this study will be approved by college of health science of Debre Markos University Research and Ethical Review Committee and if you have any question about the right of the study participant the address is:

College of health Science, Debre Markos University

P.O. Box 269 Debre Markos University, Ethiopia

Tel. 251-913-910-575

If you have question about the study the address of the principal investigator is:

Getenet Dessie (lecturer in Debre Markos University, Collage of Health Science)

Mobile: +251923522712,

E-mail: [ayalew.d16@gmail.com](mailto:ayalew.d16@gmail.com)

# ANNEX II: Individual Consent Form

I am ____________________________________ who is the data collector for a research to be conducted by Debre Markos University, College of Health Sciences.

Here at ___________ Hospital I am going to interview you to evaluate the impact of Self-Care education on self-care adherence among chronic heart failure patients. We would like to assure you your name will not be mentioned in the questionnaire and the information that you will give us will be kept confidential and only used for research purpose. You have full right to refuse to take part or to stop the interviewer at any time. But the information that you will give us is quite useful to achieve the objective of the study and to bring change on congestive heart failure patients quality of life.

Are you willing to Participate in this study? Yes No

If your response is yes sign here --------- and please continue to the next page.

**Part I: Socio-demographic and economic status characteristics of respondents**

| Se.no | Questions | Answer |
| --- | --- | --- |
| 101. | Age in Years | ­­­­____________ |
| 102 | Gender | 1.Male  2.Female |
| 103 | Hospital name | __________________ |
| 104 | Marital status | 1.Single  2.Married  3.separeted  4.Widowed  5.Divorced |
| 105. | Religion | 1. Orthodox  2. Muslim  3. Protestant  4. Catholic  5.Other (specify)____________ |
| 106. | Educational status | 1 .not able to read and write  2. primary school  3.secondar school  4. diploma  5. degree  6. Other (specify)……….. |
| 107 | Job | _____________________ |
| 108 | Main activity during the day | 1. None 2. Housework 3. Leisure 4. Others |
| 109 | Receive support from | 1. None 2. Family 3. Friends 4. Health institution |
| 110 | History of Hospitalizations | 1. Yes 2. No |
| 111 | If yes for q110, how money times have you been hospitalized | _______________________ |
| 112 | If yes for q110, how long did you stay at hospital for each hospitalization? | _______________________ |
| 113 | Weight |  |
| 114 | Height |  |

**Part II: Medical characteristics**

**Instruction:** Below there are 6 questions which tray to assess medical characteristics of the patient. Please answer each question by asking the patient and by reviewing the patient medical record.

| **Se.no** | **Questions** | **Answer** |
| --- | --- | --- |
| 200 | Duration of disease at first diagnosis | ___________years |
| 201 | Stage of CHF according to NYHA classification [check medical records] | __________ |
| 202 | Does the patient have a comorbidity (another illness) [check medical records] | 1. Yes 2. No |
| 203 | If yes for Q 202, which diseases does the patient have? | __________ |
| 204 | Previous hospital admission | 1. Yes 2. No |
| 205 | If yes for Q204, how money times was the patient hospitalized | ______________ |
| 206 | If yes for Q204, for how long did the patient stay at hospital for each hospitalization? | _______________ |
| 205 | List current medications [check medical records] | ______________ |
| 206 | Lab results [check medical records] | _______________ |

**Part III: Questions regarding to adherence for CHF self-care recommendation**

**Instruction:** Below there are 8 questions which assess your self-care practice. Please answer each question by selecting the number that best describes how often each item has been done by you in the past 1 months

| How often have you done each of the following in the past four weeks? | None of the time | A little of the time | Some of the time | A good bit of the time | Most of the time | All of the time |
| --- | --- | --- | --- | --- | --- | --- |
| Exercise regularly( at least 3 times per week) | 0 | 1 | 2 | 3 | 4 | 5 |
| Took medication as prescribed(on time without skipping doses) | 0 | 1 | 2 | 3 | 4 | 5 |
| Drank one or less alcoholic beverage per day( if you do not drink please encircle number 5) | 0 | 1 | 2 | 3 | 4 | 5 |
| Cut down on smoking or did not smoke (if you do not smoke please encircle number 5) | 0 | 1 | 2 | 3 | 4 | 5 |
| Follow a low salt diet | 0 | 1 | 2 | 3 | 4 | 5 |
| Follow a low fat diet | 0 | 1 | 2 | 3 | 4 | 5 |
| Weighed yourself every day to watch your body fluid status | 0 | 1 | 2 | 3 | 4 | 5 |
| If my leg becomes more swollen than usual, when I feel fatigue, and experienced shortness of breath, I contact doctor or nurse | 0 | 1 | 2 | 3 | 4 | 5 |

**Part IV: Questionnaire for depression screening of Congestive heart failure patient (PHQ-9)**

**Instructions**: this interview consists of nine items. Please listen carefully what we am going to read each items of statement for you, and then tell us the one number in each item that best describes the way you have been feeling during the past two weeks including today.

Depression screening questions

Not at all……………...0

Several days………..…1

More than half the days…2

Nearly every day………3

| Se  .no | Questions | Not at all | Several days | More than half days | Nearly every day |
| --- | --- | --- | --- | --- | --- |
| 1 | Little interest or pleasure in doing things |  |  |  |  |
| 2 | Feeling depressed, down or hopeless |  |  |  |  |
| 3 | Trouble falling  Or staying asleep or sleeping too much |  |  |  |  |
| 4 | Feeling tired or having little energy |  |  |  |  |
| 5 | Poor appetite or overeating |  |  |  |  |
| 6 | Feeling bad about yourself or that you are a failure or have let yourself or your family down |  |  |  |  |
| 7 | Trouble concentrating on things such as reading the newspaper or watching television |  |  |  |  |
| 8 | Moving or speaking so slowly that other people could have noticed or the opposite |  |  |  |  |
| 9 | Thoughts that you would be better off dead or of hurting yourself in some ways. |  |  |  |  |

Thanks!!!
